# Flavoromics-guided evaluation of non-Saccharomyces and Saccharomyces co-fermentation for improving the volatile aroma profile and quality of Musalais

**DOI:** 10.1016/j.fochx.2026.104694

**Published:** 2026-10-26

**Authors:** Rui Tang, Tengteng Ge, Buhailiqiemu Abudureheman, Yu Zhang, Jianlin Zhang, Lin Chen, Liang Wang, Minwei Zhang, Jingyang Hong

**Affiliations:** a Xinjiang Academy of Smart Agriculture, Urumqi 830063, China; b Musalaisi Quality Inspection Center, Xinjiang Institute of Technology, Aksu 843100,China

**Keywords:** Musalais, Non-Saccharomyces, Saccharomyces, Co-fermentation, Quality analysis, Volatile flavor compounds

## Abstract

This study evaluated the effects of co-fermentation of *Saccharomyces cerevisiae* (JD) with three *non-Saccharomyces* strains, *Debaryomyces hansenii* (HX), *Torulaspora delbrueckii* (DE), and *Schizosaccharomyces pombe* (LZ), on the fermentation characteristics, physicochemical properties, volatile metabolites, and sensory quality of Musalais. The co-fermentation groups were designated JD-H (JD + HX), JD-D (JD + DE), and JD-L (JD + LZ). Yeast biomass and fermentation behavior varied by strain, and all treatments reached the typical alcohol content range for Musalais by 96 h. Compared with monoculture, co-fermentation generally reduced alcohol and total acid contents while increasing total phenolic and flavonoid contents. A total of 1866 volatile compounds were detected, with JD-D showing the greatest difference from JD. Sensory evaluation indicated differences in aroma complexity and overall sensory quality, with scores ranked JD-L > JD-H > JD-D > JD. These findings highlight the strain-dependent effects of *non-Saccharomyces* yeasts on Musalais quality and their potential for targeted application.

## 1 Introduction

Musalais, a traditional wine from Awati County, Xinjiang, has a unique brewing process and cultural heritage involving yeast selection and volatile compound analysis (Gong et al., 2024; Tang, Abudureheman, et al., 2025; Wu et al., 2025). Current production relies mainly on pure Saccharomyces, which lacks efficacy in generating diverse aromas, resulting in simple flavor profiles without complex fruity, floral, or spicy notes (Wang, Fan, et al., 2023). Saccharomyces also poorly utilizes non-glucose carbon sources like pentoses (Ndubuisi et al., 2023).

Research has thus turned to non-Saccharomyces strains, including Torulaspora delbrueckii (Arslan et al., 2018), Schizosaccharomyces pombe (Borren & Tian, 2020), *Debaryomyces hansenii* (Delač Salopek et al., 2022), Kluyveromyces marxianus (Zhang, Xiong, et al., 2025), and Pichia anomala (Yang et al., 2023). While Saccharomyces offers strong fermentative capacity and stability (Borren & Tian, 2020), it limits flavor complexity. Co-fermentation with non-Saccharomyces strains enhances sensory quality. For example, co-fermentation with Torulaspora delbrueckii and Schizosaccharomyces pombe increased total phenols, flavonoids, and anthocyanins (Zhang, Xiong, et al., 2025); sequential fermentation with Pichia anomala improved sugar metabolism, organic acids, phenols, flavonoids, and antioxidant capacity (Zeng et al., 2025).

In preliminary studies, a superior Saccharomyces strain JD was selected from natural Musalais fermentation. Three non-Saccharomyces strains—*Debaryomyces hansenii* (ACCC20010), *Torulaspora delbrueckii* (CICC33295), and *Schizosaccharomyces pombe* (AS.2.247)—were co-fermented with JD. Debaryomyces hansenii, present in early grape juice fermentation, produces esters like isoamyl acetate and phenylethanol, enhancing floral/fruity notes (Li, Chen, et al., 2024; Xu et al., 2025). Its weaker fermentative capacity aids flavor diversity and yeast dynamics (Roca-Mesa et al., 2020). Torulaspora delbrueckii offers stable fermentation, moderate ethanol yield, increases floral/fruity compounds (e.g., isovaleric acid, phenethyl alcohol), reduces volatile acids, and improves smoothness and complexity (Wang, Huang, et al., 2024; Zhang, Wang, et al., 2024; Zhu, Torija, et al., 2021; Avîrvarei et al., 2023). *Schizosaccharomyces pombe* efficiently converts malic acid to ethanol and utilizes sucrose/fructose, benefiting fruit wines (Lyu et al., 2024). All three strains are safe, genomically characterized, and enhance flavor complexity, fermentation stability, and product consistency.

In this study, Musalais was co-fermented with *Saccharomyces* JD and three *non-Saccharomyces* yeasts at a 1:1 inoculation ratio. The resulting wines were comprehensively evaluated in terms of microbial growth, physicochemical properties, volatile flavor compounds, and sensory quality (Akbar et al., 2026; Akbar & Mustari, 2024).Volatile metabolites were analyzed using qualitative and quantitative approaches combined with principal component analysis (PCA), cluster analysis, KEGG enrichment, and flavoromics. The novelty of this study lies in the systematic comparison of three distinct *non-Saccharomyces–Saccharomyces* co-fermentation systems under the same fermentation conditions and in the integrated evaluation of yeast interactions, volatile metabolite remodeling, aroma activity, and sensory quality. This approach allows the strain-dependent effects of different *non-Saccharomyces* yeasts on Musalais fermentation and flavor formation to be elucidated, providing new insights into the relationships among microbial interactions, metabolite changes, and sensory characteristics, as well as a theoretical basis for the targeted application of mixed-culture fermentation in Musalais production.

## 2 Materials and methods

### 2.1 Materials and reagents

(1) Materials: High-quality Hetian red grapes from Aksu, Xinjiang, were selected for the fermentation process of Musalais. The yeast strains used during fermentation, *Debaryomyces hansenii* (HX, ACCC20010), *Torulaspora delbrueckii* (DE, CICC33295), and *Schizosaccharomyces pombe* (LZ, AS.2.247), were purchased from Hubei Angel Yeast Co., Ltd. in Hubei, China. *saccharomyces cerevisiae* (JD) (Tang, Abudureheman, et al., 2025) was isolated from the Musalais Testing Laboratory at the Xinjiang Institute of Technology.

(2) Reagents: Sodium bisulfite was purchased from Sichuan Jinshan Pharmaceutical Co. Ltd. (Sichuan, China), and gallic acid was purchased from Tianjin Zhonglian Chemical Reagents. (Sichuan, China), and anhydrous sodium carbonate was purchased from Tianjin Huasheng Chemical Reagent Co., Ltd. (Tianjin, China); concentrated hydrochloric acid was purchased from Guangzhou Chemical Reagents (Guangzhou, China); phenolphthalein was purchased from Tianjin Zhiyuan Chemical Reagents Co., Ltd. (Tianjin, China). Peptone, glucose, yeast extract, agar powder, and anhydrous ethanol were purchased from Beijing Aoboxing Biotechnology Co. Ltd. (Beijing, China); sodium chloride was purchased from Sinopharm Group (Beijing, China); n-hexane, which is of chromatographic grade, was purchased from Merck (Darmstadt, Germany). Unless otherwise specified, all chemical reagents and solvents were of analytical grade and used as received.

### 2.2 Experimental instruments

GC–MS/MS 8890-7000D: Agilent; Chromatographic column: DB-5MS, 30 m × 0.25 mm × 0.25 μm: Agilent; Ball mill: MM400: Retsch; Electronic balance: MS105DU: METTLER TOLEDO; Extraction head: 120 μm DVB/CWR/PDMS: Agilent; Solid-phase microextraction device: SPME Arrow: CTC Analytics AG; Aging device: Fiber Conditioning Station: CTC Analytics AG; Sample heating device: Agitator: CTC Analytics AG; Ultraviolet spectrophotometer: UV759: Shanghai Chuding Analytical Instruments Co., Ltd.; Incubator: bpH-9420: Shanghai Yiheng Scientific Instruments Co., Ltd.; Autoclave: LDZM-40KCS: Shanghai Shenan Medical Devices.

### 2.3 Methods

#### 2.3.1 Yeast counting

Yeast strains were incubated on YPD agar at 28 °C for 2 days and maintained on the same medium. Absorbance at 560 nm was measured at 0, 2, 4, 6, 8, 10, 12, 14, 16, 18, 20, 22, and 24 h, and growth curves were plotted over 24 h. Yeast cell counts in fermentation broth were determined microscopically at 24, 48, 72, 96, and 120 h.

#### 2.3.2 Musalais fermentation with *Saccharomyces* and *non-Saccharomyces* yeasts

Each yeast strain (JD, HX, DE, and LZ) was individually adjusted to 1 × 10^6^ CFU/mL with sterile water before inoculation. For the co-fermentation treatments, the JD suspension and the corresponding *non-Saccharomyces* yeast suspension were mixed and inoculated into grape juice at a 1:1 (*v*/v) ratio, thereby providing comparable initial viable cell populations of the two strains. The resulting groups were designated as JD, JD-H, JD-D, and JD-L. A pure fermentation with JD alone served as the control.

Musalais fermentation process: Based on the research by Tang, Abudureheman, et al. (2025), a direct fermentation method for Musalais was implemented with minor modifications. Hetian red grapes were selected, washed, and destemmed, then pressed to obtain grape juice. The juice was heated to 120 °C and boiled until the sugar content reached 28°Brix. After cooling, it was inoculated with a culture at 10^6^ CFU/mL (1:1 volume ratio). Fermentation was carried out at a constant temperature of 28 °C. Samples were collected at 24, 48, 72, 96, and 120 h of fermentation. Finally, the product was pasteurized at 65 °C for 30 min.

#### 2.3.3 Determination of basic physicochemical properties of Musalais

Alcohol content was determined according to GB 5009.225–2023, “National Food Safety Standard – Determination of Ethanol Content in Alcoholic Beverages and Edible Alcohols” (2023), using an alcohol meter. Reducing sugar content was determined using the anthrone‑sulfuric acid colorimetric method described by Duan et al. (2022). Total acidity was determined according to GB 12456–2021, “National Food Safety Standard – Determination of Total Acids in Foods” (2021), using the acid-base indicator method. Total phenolic content was determined using the ferric phenol reagent colorimetric method described by Zhou Zhou et al. (2023), with gallic acid used as the standard. Flavonoid content was determined according to the method described by Wu et al. (2025), with rutin used as the standard.

#### 2.3.4 Determination of volatile metabolites in Musalais

Each sample (1 mL) was placed in a headspace vial, followed by the addition of 1 mL of saturated NaCl solution and 20 μL of 1,4-di(methyl-d3)benzene‑*d*_4_ (p‑xylene‑*d*_10_; CAS 41051–88-1) as the internal standard (10 μg/mL). Volatile compounds were extracted using an automated headspace solid-phase microextraction (HS-SPME) system. Samples were equilibrated at 60 °C for 5 min, followed by headspace extraction with a 120 μm DVB/CWR/PDMS fiber for 15 min. The fiber was subsequently desorbed at 250 °C for 5 min prior to GC–MS analysis.

A DB-5MS capillary column (30 m × 0.25 mm × 0.25 μm, Agilent J&W Scientific, Folsom, CA, USA) was used for chromatographic separation. High-purity helium (≥99.999%) was used as the carrier gas at a constant flow rate of 1.2 mL/min. The injection port temperature was maintained at 250 °C, with a solvent delay of 3.5 min. The oven temperature was initially held at 40 °C for 3.5 min, increased to 100 °C at 10 °C/min, then to 180 °C at 7 °C/min, and finally to 280 °C at 25 °C/min and held for 5 min.

Mass spectrometric analysis was performed using electron impact ionization (EI) at 70 eV. The ion source, quadrupole, and interface temperatures were maintained at 230, 150, and 280 °C, respectively. A widely targeted volatilomics strategy based on selected ion monitoring (SIM) was employed. For each analyte, one quantitative ion and two to three qualitative ions were monitored in scheduled acquisition windows according to the expected retention time, enabling high-coverage detection of volatile compounds.Volatile compounds were semi-quantified using an internal-standard-based method, and the resulting values were expressed as relative contents.

#### 2.3.5 Sensory evaluation of Musalais

Sensory evaluation was conducted in accordance with GB 15038–2006 “General Analytical Methods for Wines and Fruit Wines”. The sensory panel consisted of 20 trained assessors who had received specific training in the sensory evaluation of Musalais. The samples were coded with random three-digit numbers and presented to the panelists in a randomized order so that the identities of the fermentation treatments were not disclosed during evaluation.Each panelist independently evaluated the samples in terms of appearance, aroma, taste, and aftertaste according to the sensory evaluation procedure specified in GB 15038–2006. The mean score obtained from the 20 panelists was used for subsequent analysis.

#### 2.3.6 Data processing

Volatile compounds were detected and annotated by GC–MS using a custom database. The compounds were analyzed using electron impact ionization (EI, 70 eV) and selected ion monitoring (SIM), with one quantitative ion and two to three qualitative ions monitored for each analyte in scheduled acquisition windows according to the expected retention time. Compound annotation was further supported by 10.13039/100000161NIST library matching, retention index (RI) information, mass spectral characteristics, and match factors. Similar identification strategies combining NIST database matching, mass spectral information, retention time, and match factors have been applied in recent fruit-wine metabolomics studies (Li et al., 2025), while GC–MS identification based on NIST/Wiley spectral matching and retention indices has also been reported in wine fermentation studies (Liu et al., 2025). Metabolomic data were log2-transformed, zero-centered, and processed using MassHunter. PCA was conducted with R's prcomp (center = FALSE, scale = FALSE). Cluster heatmaps used UV scaling. For pairwise comparisons, differential volatile metabolites were screened using the criteria VIP > 1 and |Log2FC| ≥ 1.0, corresponding to a fold change ≥2 or ≤ 0.5. VIP values were obtained from the OPLS-DA model. Both criteria were required simultaneously for a metabolite to be classified as differential, then annotated and enriched via KEGG.

The relative odor activity value (rOAV) of each volatile compound was calculated as the ratio of its semi-quantitative relative content to the corresponding odor threshold (rOAV = C/T), where C represents the relative content of the compound and T represents its odor threshold. Because compound contents were obtained using an internal-standard-based semi-quantitative method rather than compound-specific calibration curves, the calculated values were interpreted as relative OAVs and were used primarily to compare and rank the potential aroma contributions of volatile compounds.

All experiments were performed in triplicate. The results are presented as mean ± standard deviation (SD). Data were analyzed using Origin 2019 and SPSS 28.0, and statistical significance was determined at *P* < 0.05. The consistency among replicate measurements was evaluated based on the variation expressed as the standard deviation.

## 3 Results and discussion

### 3.1 Fermentation kinetics of yeast

Fig. 1 illustrates the fermentation kinetics of various yeast strains during Musalais fermentation. Compared with the JD monoculture, the JD-H and JD-L co-fermentation groups exhibited higher total yeast biomass, whereas the JD-D group showed comparatively lower total biomass. These results indicate that different non-Saccharomyces strains can produce distinct effects on the overall population dynamics of mixed fermentation systems, consistent with previous reports that yeast interactions in mixed cultures are strain-dependent (Wang et al., 2025). Similar strain-dependent effects have also been reported in other non-Saccharomyces/Saccharomyces co-fermentation systems. For example, Li, Huang, et al. (2024) observed that the introduction of T. delbrueckii and *H. uvarum* altered the population dynamics of *S. cerevisiae* during mixed fermentation, indicating that the interactions among different yeast species can vary substantially depending on the strain combination. In comparison, the present study showed that JD-H and JD-L resulted in higher total yeast biomass than JD, whereas JD-D resulted in lower biomass, further supporting the strain-dependent nature of yeast population changes during co-fermentation. However, because the cell counts represented the total yeast population in each mixed culture and species-specific populations were not separately quantified, the present results do not provide direct evidence of synergistic or competitive interactions between JD and the individual non-Saccharomyces strains.

**Fig. 1:**
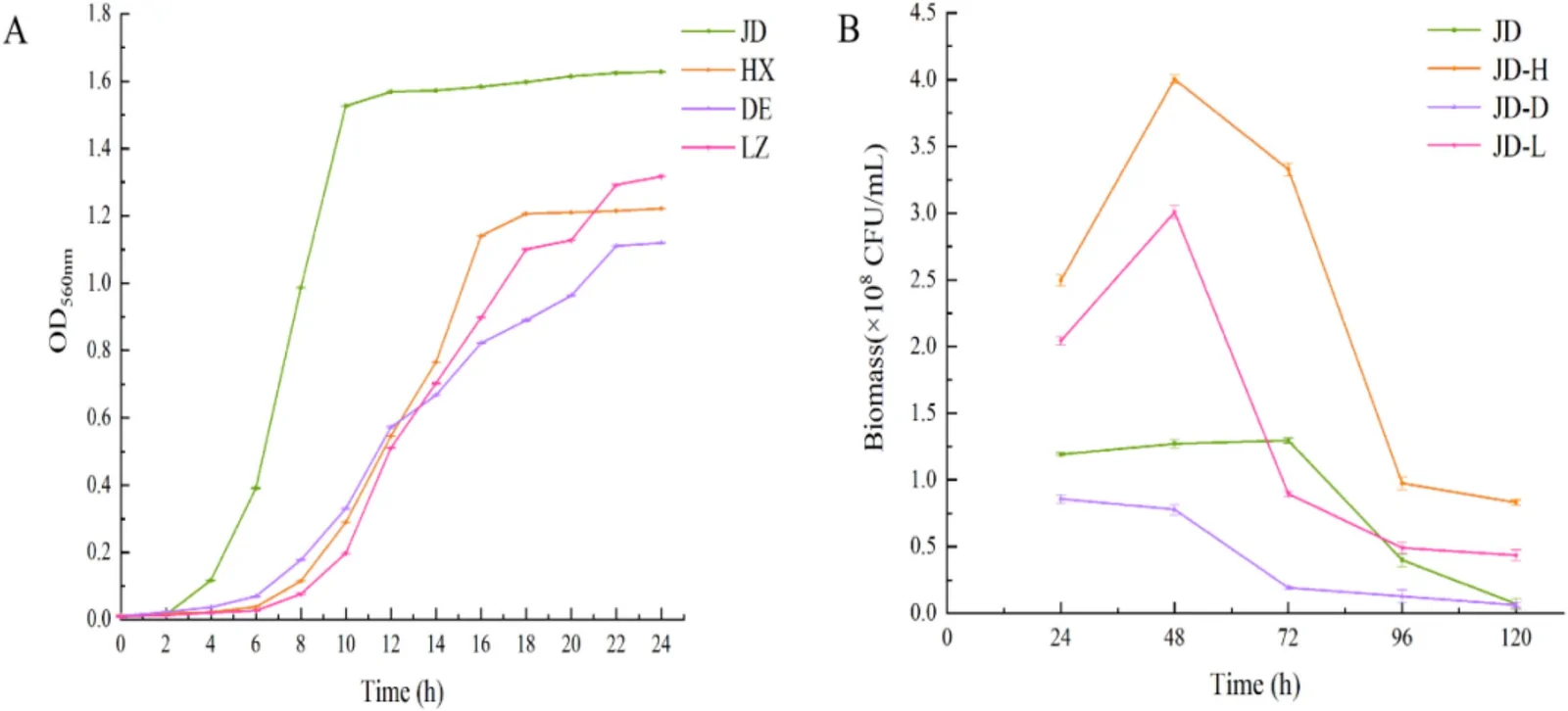
Fermentation kinetics curve of yeast

### 3.2 Analysis of changes in the basic physicochemical properties of Musalais

Changes in key physicochemical parameters during Musalais fermentation are shown in Table 1. The alcohol content serves as an indicator of both alcohol yield and substrate utilization efficiency. Alcohol is generated through yeast-mediated conversion of fermentable grape sugars (Listur et al., 2026). As fermentation proceeds, the rising alcohol content contributes to the flavor balance and stability of Musalais (Del Bosque et al., 2026). Therefore, variations in alcohol content can serve as key indicators of the fermentation stage of Musalais. As presented in Table 1, the alcohol content in all fermentation groups increased over time. According to the Musalais standard (DB 65/T 2924–2008), the typical alcohol content range is 8.0–14.0% (*v*/v). By 96 h, the alcohol contents of all fermentation groups had reached this typical range. However, ethanol concentrations continued to increase between 96 and 120 h, indicating that alcoholic fermentation had not yet been fully completed at 96 h. At 120 h, the alcohol content of the JD group increased from 13.47% to 17.00% (*v*/v), while that of the JD-L group reached 16.93% (*v*/v), both exceeding the typical range specified for Musalais. These results indicate that extending fermentation to 120 h may lead to excessive ethanol accumulation and may therefore be unsuitable as a practical fermentation endpoint. In contrast, the alcohol contents of all groups remained within the typical range at 96 h. Therefore, 96 h may represent a more appropriate practical endpoint for controlling alcohol content, although alcoholic fermentation was still ongoing at this stage. Compared with JD monoculture, the co-fermentation treatments generally showed lower ethanol concentrations at the later fermentation stages, indicating that the addition of non-Saccharomyces yeasts altered ethanol production in a strain-dependent manner.Similarly, Arslan et al. (2018) reported lower ethanol production in T. delbrueckii–*S. cerevisiae* co-fermentation than in *S. cerevisiae* monoculture. In the present study, however, the effect varied among non-Saccharomyces strains, with JD-H and JD-D showing lower final alcohol contents than JD-L.

**Table 1:** Changes in basic physical and chemical parameters during the fermentation process by Musalais.

Fermentation Group	JD	JD-H	JD-D	JD-L
Alcohol content (%*v*/v)				
24 h	7.00 ± 0.03^a^	5.33 ± 0.02^c^	6.13 ± 0.02^b^	7.07 ± 0.01^a^
48 h	8.27 ± 0.01^b^	5.93 ± 0.02^d^	7.00 ± 0.01^c^	8.87 ± 0.02^a^
72 h	10.04 ± 0.01^a^	7.53 ± 0.06^c^	8.93 ± 0.02^b^	10.37 ± 0.05^a^
96 h	13.47 ± 0.06^a^	12.13 ± 0.02^b^	11.05 ± 0.01^c^	13.43 ± 0.04^a^
120 h	17.00 ± 0.04^a^	14.87 ± 0.02^b^	14.57 ± 0.06^c^	16.93 ± 0.02^a^

Reducing sugars (g/L)				
24 h	78.90 ± 0.08^a^	80.36 ± 0.08^a^	79.57 ± 0.01^a^	82.08 ± 0.08^a^
48 h	68.67 ± 0.05^c^	77.88 ± 0.07^a^	74.49 ± 0.06^b^	71.89 ± 0.07^bc^
72 h	67.38 ± 0.07^a^	44.20 ± 0.05^c^	59.73 ± 0.02^b^	69.64 ± 0.09^a^
96 h	50.38 ± 0.01^a^	18.26 ± 0.09^c^	38.97 ± 0.08^b^	48.86 ± 0.04^a^
120 h	32.86 ± 0.05^a^	9.41 ± 0.08^d^	21.09 ± 0.08^c^	26.71 ± 0.08^b^

Total acidity (g/L)				
24 h	4.73 ± 0.01^a^	4.73 ± 0.08^a^	4.47 ± 0.08^a^	4.73 ± 0.05^a^
48 h	5.45 ± 0.01^a^	4.78 ± 0.08^b^	4.91 ± 0.08^b^	4.73 ± 0.05^b^
72 h	5.54 ± 0.07^a^	5.09 ± 0.04^a^	4.91 ± 0.08^a^	5.09 ± 0.04^a^
96 h	5.72 ± 0.01^a^	5.27 ± 0.06^b^	5.23 ± 0.04^b^	5.18 ± 0.08^b^
120 h	6.24 ± 0.07^a^	5.36 ± 0.02^b^	5.32 ± 0.08^b^	5.27 ± 0.08^b^

Total phenols (g/L)				
24 h	0.72 ± 0.01^c^	0.70 ± 0.04^b^	0.66 ± 0.05^b^	0.89 ± 0.06^a^
48 h	0.73 ± 0.02^b^	0.80 ± 0.02^b^	0.82 ± 0.06^b^	0.90 ± 0.05^a^
72 h	0.87 ± 0.03^a^	0.88 ± 0.28^a^	0.92 ± 0.07^a^	0.96 ± 0.02^a^
96 h	0.96 ± 0.05^c^	1.07 ± 0.03^b^	1.32 ± 0.03^a^	1.06 ± 0.02^b^
120 h	0.71 ± 0.02^d^	0.92 ± 0.04^c^	1.16 ± 0.04^a^	0.99 ± 0.03^b^

Flavonoids (g/L)				
24 h	0.28 ± 0.03^c^	0.28 ± 0.02^c^	0.34 ± 0.04^b^	0.44 ± 0.03^a^
48 h	0.35 ± 0.03^c^	0.32 ± 0.03^c^	0.41 ± 0.04^b^	0.50 ± 0.01^a^
72 h	0.59 ± 0.04^a^	0.40 ± 0.02^b^	0.46 ± 0.07^b^	0.62 ± 0.03^a^
96 h	0.70 ± 0.07^b^	0.46 ± 0.02^c^	0.49 ± 0.01^c^	0.86 ± 0.04^a^
120 h	0.43 ± 0.02^b^	0.44 ± 0.04^b^	0.44 ± 0.02^b^	0.65 ± 0.03^a^

**Note:** Values represented by different lowercase letters in the same row are significantly different (*P* < 0.05).

Reducing sugars serve as the primary substrates for alcoholic fermentation, directly influencing both the alcohol yield and the residual sweetness of the wine. Throughout fermentation, these sugars are progressively metabolized by yeast and converted into ethanol, carbon dioxide, and other compounds associated with flavor development (Li, Yuan, et al., 2024). The observed decrease in reducing sugar content across all groups over time reflects the ongoing conversion of substrates into alcohol and other metabolic products.Reducing sugars serve as the primary carbon substrates for alcoholic fermentation and are progressively consumed during fermentation. As shown in Table 1, reducing sugar concentrations decreased in all fermentation groups over time. After 120 h, the JD group retained 32.86 ± 0.05 g/L reducing sugars, whereas the JD-H group contained only 9.41 ± 0.08 g/L, indicating substantially greater sugar utilization in the JD-H treatment. Notably, this greater depletion of reducing sugars was not accompanied by a correspondingly higher ethanol concentration. This pattern suggests that carbon utilization in the JD-H co-fermentation system differed from that in the JD monoculture, with a greater proportion of the consumed carbon potentially being redirected toward other fermentation-associated products and cellular processes, such as organic acid formation, glycerol production, biomass accumulation, and secondary metabolite biosynthesis. Such carbon redistribution may also contribute to the differences observed in the physicochemical and volatile metabolite profiles of the co-fermentation groups. Arslan et al. (2018) also reported altered sugar utilization and lower ethanol production in T. delbrueckii–*S. cerevisiae* co-fermentation. Consistently, JD-H showed greater reducing-sugar consumption but lower ethanol production than JD in the present study, indicating strain-dependent differences in carbon utilization.

Total acids are the key components responsible for the acidic taste of Musalais. These organic acids not only help balance the sweetness and alcohol content of Musalais, but also contribute to the formation of various flavor compounds. Some of these acids are directly synthesized by yeast. During fermentation, the total acid content increased gradually. In the JD group, acid accumulation occurred most rapidly and reached the highest level, attaining 6.24 ± 0.07 g/L after 120 h. This group also exhibited the highest concentrations of acidic metabolites, leading to a more pronounced acidity. By contrast, the total acid content in the three fermentation groups using non-Saccharomyces was significantly lower, resulting in a milder acidic profile.Similar effects have been reported in other mixed-yeast fermentations. Arslan et al. (2018) and Liu et al. (2022) reported altered or reduced acid accumulation following T. delbrueckii co-fermentation, consistent with the lower total acidity observed in the non-Saccharomyces groups in the present study.

Total phenols are primarily derived from the skins, seeds, and pulp of grapes. These compounds play a crucial role in providing antioxidant activity, color, and astringency to Muscat grapes. Throughout fermentation, a portion of these phenols is extracted and converted into other substances. The total phenol concentration peaked during the mid-fermentation stage, after which it declined slightly, likely due to oxidation or polymerization processes. The total phenol content is positively correlated with antioxidant capacity (Lin et al., 2023). As phenolic constituents, flavonoids significantly influence the quality, health benefits, and storage stability of Musalais (Xue et al., 2022). According to Table 1, both total phenol and flavonoid contents increased initially, peaking at 96 h of fermentation, before subsequently declining. The addition of different non-Saccharomyces yeasts affected the phenolic composition in a strain-dependent manner. The JD group showed comparatively lower total phenol levels than the co-fermentation treatments. At 24 h of fermentation, flavonoid concentrations were lowest in the JD and JD-H groups (0.28 ± 0.03 g/L). By 96 h, the JD-L group exhibited the highest flavonoid level (0.86 ± 0.04 g/L), and its flavonoid content remained comparatively high throughout the fermentation period. This observation indicates an association between JD-L co-fermentation and increased flavonoid content; however, the specific biological mechanism responsible for this increase was not directly investigated in the present study. Therefore, the higher flavonoid content observed in JD-L should be interpreted as a strain-dependent fermentation effect rather than as evidence of a specific metabolic mechanism of S. pombe. Similarly, Zhang, Xiong, et al. (2025) reported that different non-Saccharomyces yeasts affected the phenolic and flavonoid contents of strawberry wine. In the present study, JD-L showed the highest flavonoid content at 96 h, further indicating a strain-dependent effect of non-Saccharomyces yeasts on phenolic profiles.

In contrast, the JD group showed the lowest flavonoid content at 120 h. A potential explanation for the decline in flavonoid levels at the later fermentation stage is that clarification and filtration may have removed certain flavonoid–protein complexes associated with colloidal particles, thereby lowering the overall flavonoid content in the final product (Pintać Šarac et al., 2024).

### 3.3 Analysis of volatile flavors in Musalais

Fig. 2 presents a circular diagram illustrating the metabolite categories identified in Musalais. A total of 1866 volatile flavor compounds were detected, falling into 15 distinct categories. The major categories comprised esters (20.2%), terpenoids (17.58%), ketones (10.77%), heterocyclic compounds (10.45%), alcohols (8.04%), acids (5.63%), aldehydes (5.63%), phenols (5.25%), hydrocarbons (4.5%), amines (3.86%), aromatic compounds (2.84%), ethers (2.41%), nitrogen-containing compounds (1.66%), halogenated hydrocarbons (0.59%), and sulfur-containing compounds (0.59%). Among these, esters, terpenoids, ketones, and heterocyclic compounds were the most abundant. Approximately 45% of these compounds are volatile flavor substances generated through the fermentation of various yeast strains in Musalais wine. Collectively, volatile flavor substances constitute 59% of all flavor compounds in Musalais. Esters, which are primarily synthesized by yeast during alcohol fermentation, represent significant flavor components in Musalais. They contribute to the fruity and floral notes in Musalais (Li et al., 2023). Terpenes are also key determinants of grape and wine flavor and quality, typically imparting floral aromas, such as rose, citrus, and honey, along with plant-like scents (Mele et al., 2020). Ketones play a notable role in wine flavor by enhancing sweet and smoky nuances (Marchand et al., 2000). Although heterocyclic compounds occur in wine at low concentrations, they can markedly shape the flavor profile, adding notes of caramel, nuts, roast, or earthiness (Shi et al., 2024). In the Musalais examined here, however, heterocyclic compounds accounted for a relatively high proportion (10.45%), likely a result of the boiling process used in preparing the fermentation broth.

**Fig. 2:**
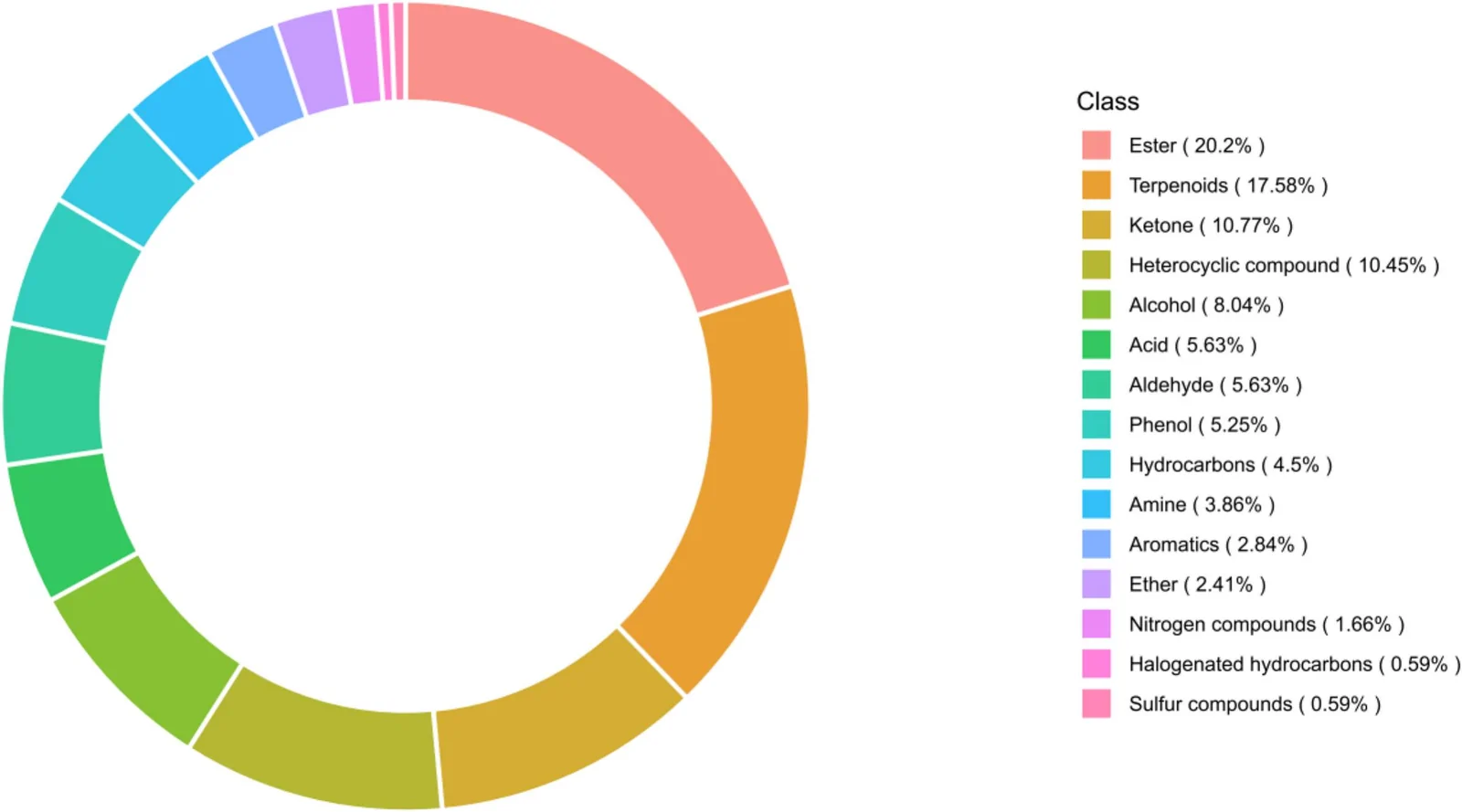
Circular diagram of metabolite categories.

Fig. 3 presents a radar chart depicting the mass concentrations of volatile flavor compounds in Musalais (for compounds with concentrations >1). The chart illustrates the relative differences in the contents of major volatile compound classes among the four fermentation groups, including alcohols (150 types), amines (72 types), esters (377 types), ketones (201 types), terpenes (328 types), aldehydes (105 types), and nitrogen-containing compounds (31 types). Alcohols, esters, ketones, and aldehydes are important contributors to the fermentation- and aging-related flavor characteristics of Musalais (Yu et al., n.d.). As illustrated in Fig. 3, the JD-D group exhibited the greatest overall abundance of volatile flavor compounds among the fermentation groups, followed by the JD-L group. These findings indicate that the effects of co-fermentation on volatile metabolite profiles were strain-dependent (Lian et al., 2024; Wang, Fu, et al., 2024). Compared with JD monoculture, co-fermentation with DE and LZ markedly altered the relative abundance of several major volatile compound classes, including alcohols, esters, and terpenoids. These changes suggest that different non-Saccharomyces strains can modulate flavor formation in distinct ways during Musalais fermentation. Although the concentrations of volatile flavor compounds varied among the four fermentation groups, the major compound classes detected were broadly similar. In all groups, alcohols, esters, and aldehydes represented important components of the overall volatile profile. Similarly, Li, Chen, et al. (2024) and Wang, Fu, et al. (2024) reported that non-Saccharomyces co-fermentation altered esters, higher alcohols, and terpenes. In the present study, JD-D showed the highest overall volatile abundance, whereas JD-L showed a distinct volatile profile, further supporting the strain-dependent effects of co-fermentation.Overall, these results suggest that co-fermentation primarily affected the relative abundance of shared volatile compound classes rather than fundamentally altering the overall composition of the volatile. Profile.

**Fig. 3:**
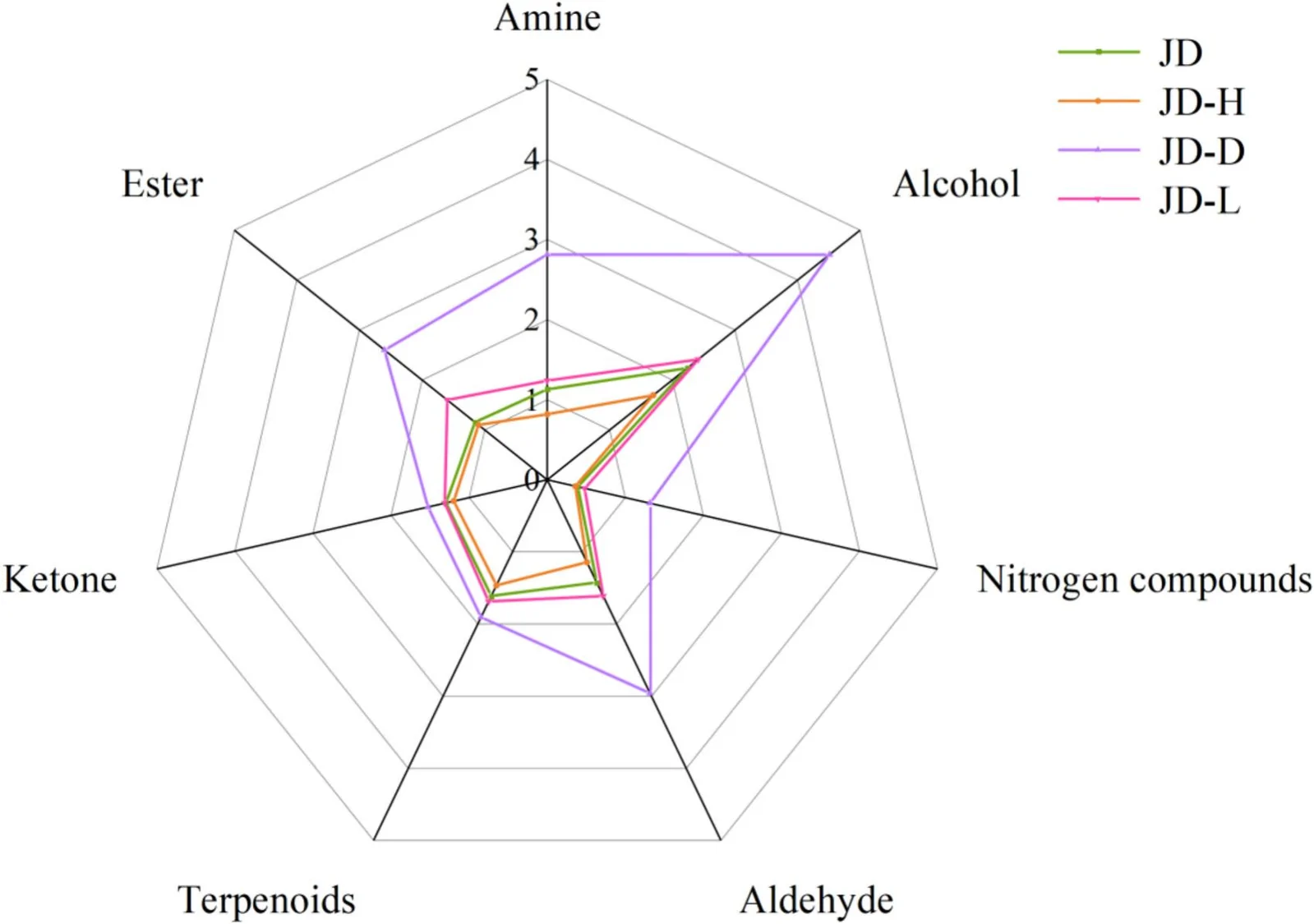
Radar chart of the mass concentration of volatile flavor compounds in Musalais (unit: μg/mL).

### 3.4 Analysis of differences between different fermentation groups

Venn diagrams depict the overlap and occurrence patterns of volatile compounds across the four fermentation treatments. As presented in Fig. 4, a total of 1866 volatile compounds were detected and annotated in Musalais, of which 1826 were common to all four fermentation groups, accounting for approximately 97.9% of the detected compounds. This indicates that the overall volatile composition was highly conserved among the treatments. The remaining 40 compounds showed non-universal occurrence patterns and were detected in two or three of the four fermentation groups rather than being uniquely present in a single treatment. In contrast to the increased volatile diversity reported for *S. cerevisiae*–S. pombe co-fermentation in mandarin wine (Luo et al., 2023), 97.9% of the volatile compounds detected in the present study were shared among all fermentation groups. This suggests that non-Saccharomyces co-fermentation mainly altered the relative abundance of shared volatile compounds rather than generating a distinct volatile compound pool.

**Fig. 4:**
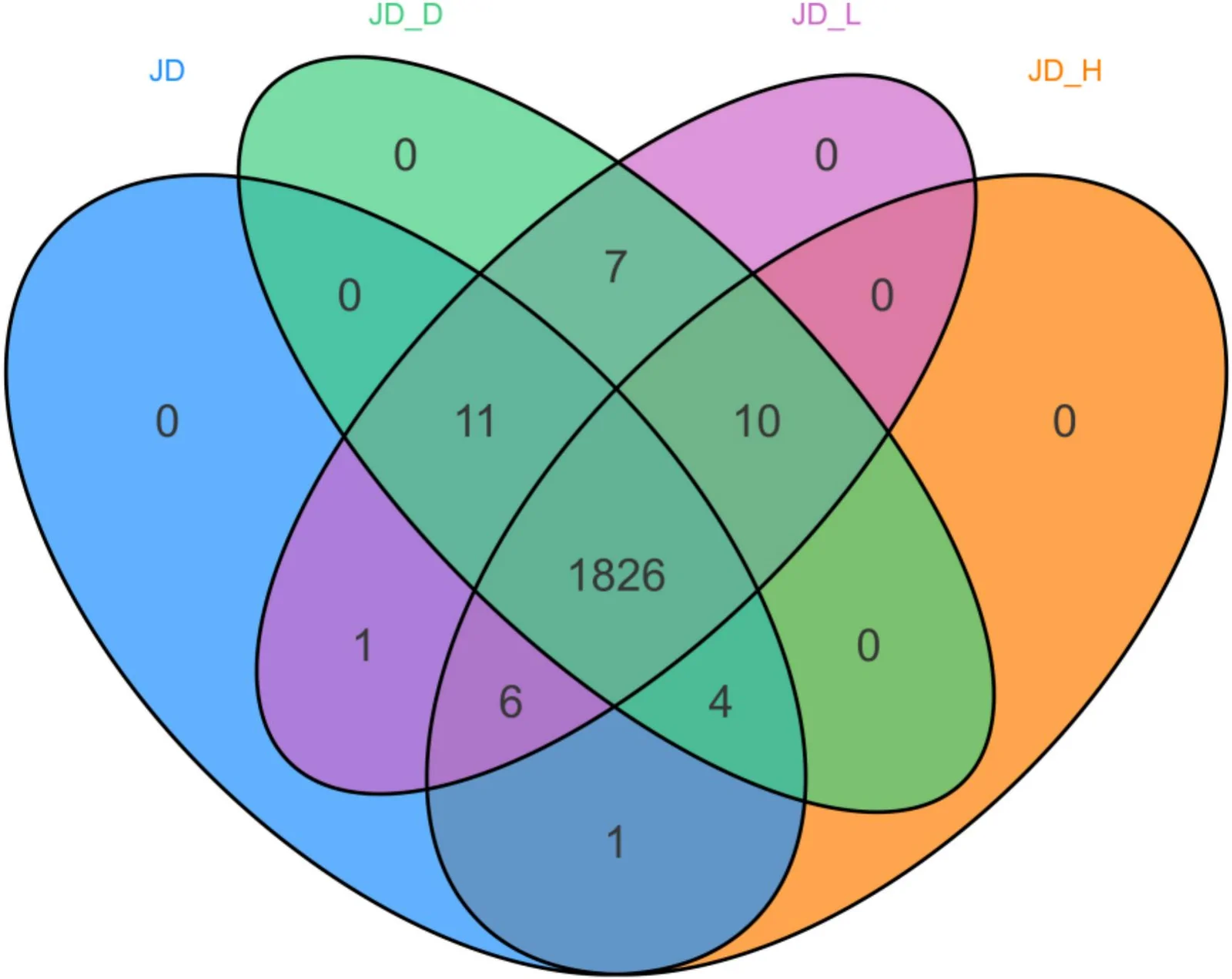
Venn diagram comparing different fermentation groups.

The identities, chemical classes, CAS numbers, retention indices, and occurrence patterns of these compounds are provided in Supplementary Table S3. These results suggest that the major effects of co-fermentation were reflected primarily in quantitative differences among the shared volatile compounds rather than in the appearance or disappearance of large numbers of compounds. Therefore, intergroup differences were further evaluated using multivariate statistical analyses and differential metabolite screening.

Principal component analysis (PCA) was performed to visualize the overall variation in volatile metabolite profiles among the four fermentation groups. As shown in Fig. 5-A, PC1 and PC2 explained 44.94% and 11.85% of the total variance, respectively, accounting for a cumulative 56.79%. Therefore, the two-dimensional PCA score plot captured a substantial but incomplete proportion of the overall variation, and the apparent separation among groups should be interpreted with caution. As shown in Fig. 5-B, PC3 explained an additional 11.24% of the variance, increasing the cumulative explained variance of the first three principal components to 68.03%. The three-dimensional PCA plot therefore provided additional information on the overall distribution of the samples. Taken together, the PCA results suggested differences in volatile metabolite profiles among the fermentation groups, with JD-D showing the most distinct distribution relative to JD, whereas JD-H exhibited a comparatively similar pattern to JD. These PCA observations were subsequently considered together with the differential metabolite analyses to further evaluate intergroup differences.

**Fig. 5:**
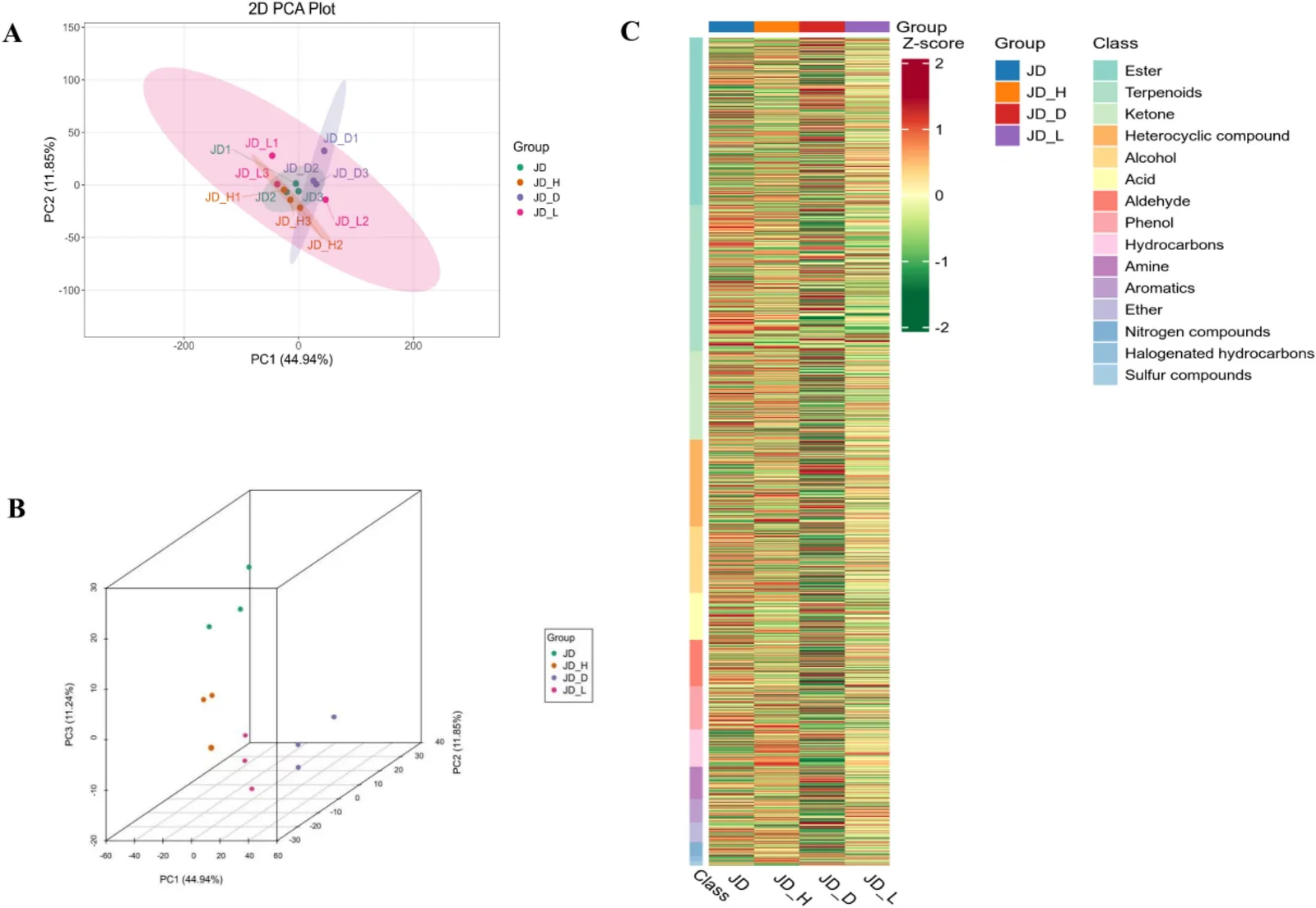
PCA score plots and overall clustering diagram for the 4 fermentation groups.

The overall clustering pattern of the four fermentation groups is presented in Fig. 5-C. The horizontal axis denotes the sample names, while the vertical axis corresponds to volatile flavor compounds. On the left side, the dendrogram illustrates the clustering of these compounds, and the dendrogram at the top reflects the clustering of samples. Colors indicate normalized values based on relative content, with red representing high content and green indicating low content. From the diagram, it can be observed that the JD-H group displays a pattern similar to that of the JD group, with only minor variations in certain regions, suggesting minimal differences between these two groups. In contrast, the JD-D group exhibited multiple red areas, reflecting substantially higher levels of characteristic flavor compounds. This group demonstrated the most pronounced differences among the four groups, which aligns with the PCA findings. The JD-L group shared several high-content compounds with the JD-D group, while in other regions its pattern resembled those of the JD and JD-H groups.Overall, JD-D showed the most distinct volatile metabolite profile relative to JD, followed by JD-L, whereas JD-H exhibited a comparatively similar pattern to JD.

### 3.5 Differential volatile metabolites between co-fermentation treatments and JD monoculture

The volcano plot presented in Fig. 6 illustrates differentially expressed compounds based on Log2(Fold Change) and VIP scores. This visualization facilitates the comparison of compound expression patterns across groups. Each point corresponds to an individual compound: red denotes upregulated compounds, green indicates downregulated compounds, and gray represents compounds detected without significant intergroup differences. The horizontal axis reflects the magnitude of relative concentration differences between groups, with larger absolute values indicating greater disparities. The vertical axis corresponds to VIP values. Higher VIP values indicate a greater contribution of individual compounds to group discrimination in the OPLS-DA model. As illustrated in Fig. 6-A, 48 upregulated and 44 downregulated compounds were detected in the JD and JD-H groups, respectively. In Fig. 6-B, 263 upregulated and 91 downregulated compounds were identified in the JD and JD-D groups, respectively. Fig. 6-C shows 75 upregulated and 83 downregulated compounds in the JD and JD-L groups, respectively. The order of significance for the intergroup differences was as follows: JD-D vs. JD group > JD-L vs. JD group > JD-H vs. JD group.

**Fig. 6:**
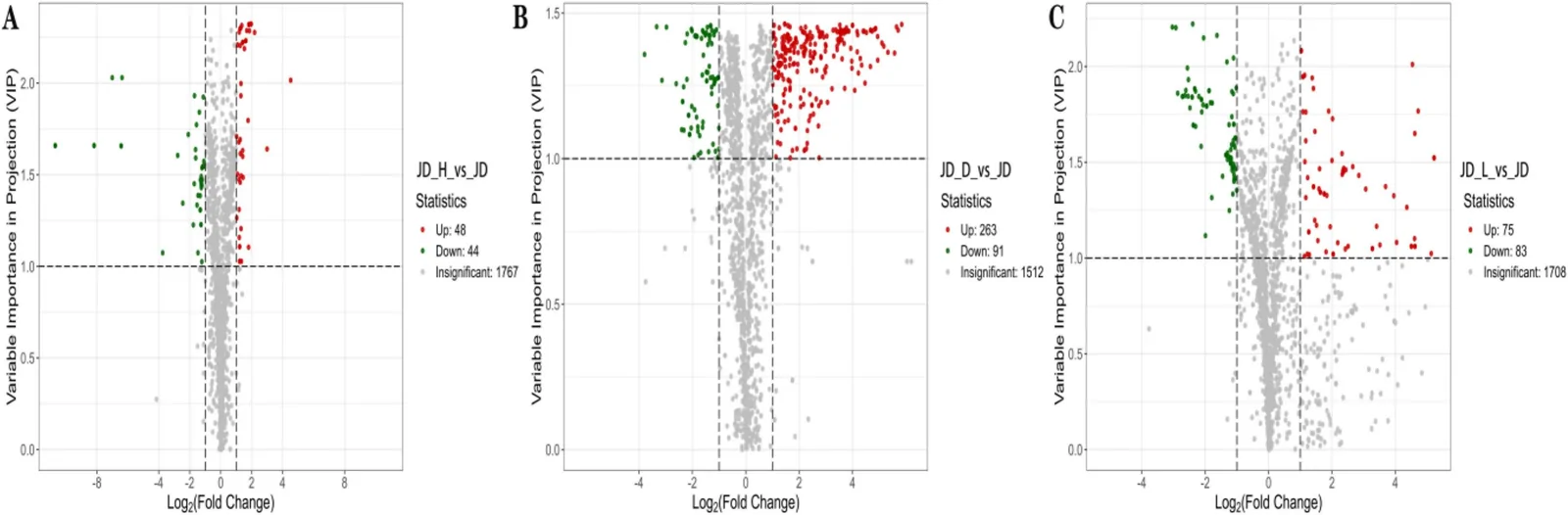
Volcanic diagram of Musalais's isomeric compounds.

Based on the screening criteria of VIP > 1 and |Log2FC| ≥ 1.0, the top 40 differential volatile compounds were selected for further comparison. As presented in Table 2, compared to the JD group, the JD-H group contained two amines, one aromatic compound, two ethers, five aldehydes, two acids, eight terpenes, two hydrocarbons, five ketones, seven heterocyclic compounds, and six esters. Among these compounds, the most pronounced increase was observed in 2-oxooctanoic acid, with its concentration in the JD-H group reaching approximately 22.9 times that in the JD group. In contrast, the most marked decrease was noted for the terpene compound 1R,4R,7R,11R-1,3,4,7-tetramethyltricyclo[5.3.1.0(4,11)]undecyl-2-ene, which was present in the JD-H group at only 0.47 times the level measured in the JD group. 2-Oxooctanoic acid generally functions as an intermediate in the metabolic pathways of various organisms and also acts as a precursor for numerous volatile flavor compounds generated during Maillard and other thermal reactions (Zheng et al., 2023). These compounds contribute to the flavor profile of Musalais. A number of pyrazine compounds were identified (Table 2), such as 2-ethyl-5-methylpyrazine, 2-ethyl-6-methylpyrazine, and 2-ethyl-3-methylpyrazine. These compounds, which are linked to Maillard reactions during thermal processing, enhance the roasted and nutty notes characteristic of Musalais (Du et al., 2024; Wang, Fan, et al., 2023). Liu et al. (2022) and Wang, Huang, et al. (2024) also reported significant changes in individual esters and other volatile compounds during non-Saccharomyces co-fermentation. Consistently, the present study showed marked strain-dependent changes in specific volatiles, including a 57.31-fold increase in butyl 3-methylbutyrate in JD-D and an increase in 3-carene in JD-L. These changes indicate strain-dependent modulation of volatile profiles rather than a confirmed biosynthetic mechanism.

**Table 2:** Top 40 compounds showing differences in volatile flavor compounds between the JD and JD-H groups.

Compounds	Class I	VIP	Fold_Change	Log2FC	Type
2-Oxooctanoic acid	Acid	2.0156	22.9176	4.5184	up
Ethanone, 1-(4-ethylphenyl)-	Ketone	1.6404	7.9584	2.9925	up
Hexanoic acid, 2-ethyl-	Acid	2.2764	4.5466	2.1848	up
Benzene, 1-methoxy-4-methyl-2-(1-methylethyl)-	Ether	2.3226	3.9013	1.9639	up
Benzene, 2-methoxy-4-methyl-1-(1-methylethyl)-	Terpenoids	2.3226	3.9013	1.9639	up
1-(5-Dimethylethyl)pyrazin-2-yl-ethan-1-one	Ketone	2.2853	3.6077	1.8511	up
Pyrazine, 2-ethyl-5-methyl-	Heterocyclic compound	2.3198	3.5244	1.8174	up
Pyrazine, 2-ethyl-6-methyl-	Heterocyclic compound	2.3198	3.5244	1.8174	up
Pyrazine, 2-ethyl-3-methyl-	Heterocyclic compound	2.3198	3.5244	1.8174	up
Butanoic acid, 2-butoxy-1-methyl-2-oxoethyl ester	Ester	1.1042	3.4758	1.7973	up
1H-Pyrrole-2-carboxaldehyde	Aldehyde	1.7962	3.3984	1.7649	up
Ethanone, 1-(2,4-dimethyl-1H-pyrrol-3-yl)-	Ketone	2.2867	3.2053	1.6804	up
Pyrazine, 3,5-diethyl-2-methyl-	Heterocyclic compound	2.2315	3.0749	1.6205	up
2,3,5-Trimethyl-6-ethylpyrazine	Heterocyclic compound	2.2315	3.0749	1.6205	up
Cyclohexane, propyl-	Hydrocarbons	2.1883	2.8833	1.5277	up
2-Cyclopenten-1-one, 3-methyl-2-(2-pentenyl)-, (*Z*)-	Ketone	1.4852	2.7225	1.4449	up
1-Pentadecene	Hydrocarbons	1.5999	2.6714	1.4176	up
trans-11-Tetradecenyl acetate	Ester	1.6321	2.6702	1.4170	up
Pyrazine, 2-ethyl-3,5-dimethyl-	Heterocyclic compound	2.2221	2.6696	1.4166	up
Pyrazine, 2,6-diethyl-	Heterocyclic compound	2.2221	2.6696	1.4166	up
1R,4R,7R,11R-1,3,4,7-Tetramethyltricyclo[5.3.1.0(4,11)]undec-2-ene	Terpenoids	1.5734	0.4701	−1.0889	down
3-Cyclohexene-1-methanol, .alpha.,.alpha.,4-trimethyl-, (*R*)-	Terpenoids	1.5451	0.4642	−1.1072	down
1-Undecanamine	Amine	1.9240	0.4584	−1.1254	down
2-Propenal, 3-phenyl-	Aldehyde	1.5444	0.4450	−1.1681	down
Cinnamaldehyde, (*E*)-	Aldehyde	1.5444	0.4450	−1.1681	down
2-Heptanol, acetate	Ester	1.4730	0.4360	−1.1977	down
Propyl octanoate	Ester	1.5372	0.4309	−1.2147	down
Benzeneacetaldehyde	Aldehyde	1.4431	0.4290	−1.2208	down
Dihydroxyacetone	Ketone	1.0252	0.4286	−1.2224	down
3-Hexene, 1-(1-methoxyethoxy)-, (*Z*)-	Ether	1.4603	0.4211	−1.2478	down
Benzeneacetaldehyde, .alpha.-ethylidene-	Aldehyde	1.4292	0.4190	−1.2550	down
Benzoic acid, 1,2,3,4,5-pentamethylcyclopenta-2,4-dienyl ester	Ester	1.4421	0.4185	−1.2566	down
DL-Norvaline, ethyl ester	Ester	1.4909	0.4176	−1.2598	down
Indene	Aromatics	1.4539	0.4166	−1.2631	down
trans-.alpha.-Bergamotene	Terpenoids	1.2277	0.4127	−1.2767	down
cis-.alpha.-Bergamotene	Terpenoids	1.2277	0.4127	−1.2767	down
Pentanamide	Amine	1.4710	0.4011	−1.3180	down
1,3,6-Octatriene, 3,7-dimethyl-, (Z)-	Terpenoids	1.3081	0.4000	−1.3220	down
.beta.-Ocimene	Terpenoids	1.3081	0.4000	−1.3220	down
1,3,7-Octatriene, 3,7-dimethyl-	Terpenoids	1.3081	0.4000	−1.3220	down

As indicated in Table 3, a comparison between the JD and JD-D groups revealed 40 compounds with significant differences. These comprised two amines, six alcohols, one halogenated hydrocarbon, one acid, nine terpenoids, eleven hydrocarbons, three ketones, six heterocyclic compounds, and eleven esters. Among them, the most pronounced increase was observed for butyl 3-methylbutyrate, an ester compound. In the JD-D group, the level was elevated by 57.3-fold compared to the JD group. The most pronounced reduction was noted for 2-decanone, a ketone compound, which decreased to 0.5 times the level in the JD group. Butyl 3-methylbutyrate imparts a characteristic fruity and sweet aroma reminiscent of bananas, apples, and tropical fruits (Goulioti et al., 2023; Xiao et al., 2024). It imparts a pleasant, fresh, and fruity aroma to Musalais, increasing the overall complexity and appeal of the fragrance. With its creamy and citrus notes accompanied by a subtle waxy character (Sansone-Land et al., 2014), 2-decanone contributes to a smoother, more delicate taste while adding depth to the flavor profile of Musalais.

**Table 3:** Top 40 compounds showing differences in volatile flavor compounds between the JD and JD-D groups.

Compounds	Class I	VIP	Fold_Change	Log2FC	Type
Butanoic acid, 3-methyl-, butyl ester	Ester	1.4610	57.3143	5.8408	up
2-Heptanol, 5-ethyl-	Alcohol	1.4415	52.1992	5.7060	up
Butanoic acid, 1-phenylethyl ester	Ester	1.4525	50.0826	5.6462	up
1,9-Nonanedithiol	Alcohol	1.3625	47.5571	5.5716	up
Butanoic acid, decyl ester	Ester	1.3654	40.0824	5.3249	up
Butyl caprate	Ester	1.3863	35.9796	5.1691	up
N-(4-Methylphenyl)-2-diazo-2-cyano-acetamide	Amine	1.3972	34.3045	5.1003	up
Benzenecarbothioamide	Amine	1.3382	30.7503	4.9425	up
Octadecane, 1-chloro-	Halogenated hydrocarbons	1.4195	30.6765	4.9391	up
Indole, 3-methyl-	Heterocyclic compound	1.4468	29.1238	4.8641	up
1H-Indole, 2-methyl-	Heterocyclic compound	1.4468	29.1238	4.8641	up
2-methyl-2-butenoic *aci* phenethyl ester	Ester	1.4436	28.1890	4.8171	up
Dodecanoic acid, ethyl ester	Ester	1.4379	26.5186	4.7289	up
2-Naphthalenemethanol	Alcohol	1.4412	25.5799	4.6769	up
1-Naphthalenemethanol	Alcohol	1.4412	25.5799	4.6769	up
6-Methyl-2-(piperidin-1-ylmethyl)-4-pyrimidinylamine	Heterocyclic compound	1.3165	25.4365	4.6688	up
Guaiol	Terpenoids	1.4331	22.9580	4.5209	up
Metronidazole	Alcohol	1.2593	22.1144	4.4669	up
Isoquinoline, 1-butyl-3,4-dihydro-	Heterocyclic compound	1.4375	21.6020	4.4331	up
Cetene	Hydrocarbons	1.4378	21.1022	4.3993	up
2-Decanone	Ketone	1.0332	0.4984	−1.0045	down
4-Methylpentyl 2-methylbutanoate	Ester	1.1049	0.4981	−1.0056	down
Butanoic acid, 2-methyl-, propyl ester	Ester	1.2788	0.4917	−1.0242	down
Ethanone, 1-(4-hydroxy-3-thienyl)-	Ketone	1.0035	0.4911	−1.0259	down
Triethyl phosphate	Ester	1.1819	0.4853	−1.0429	down
2,5-Furandione, dihydro-3-methyl-	Ketone	1.0041	0.4848	−1.0445	down
2-Ethyl-3-methoxypyrazine	Heterocyclic compound	1.2764	0.4797	−1.0599	down
3-Methoxy-2,5-dimethylpyrazine	Heterocyclic compound	1.2764	0.4797	−1.0599	down
4-Methyl-2-oxovaleric acid	Acid	1.4424	0.4667	−1.0995	down
Acetoacetic acid isoamyl ester	Ester	1.0254	0.4549	−1.1364	down
Acetic acid, cyano-, methyl ester	Ester	1.2639	0.4479	−1.1587	down
isoledene	Terpenoids	1.2708	0.4471	−1.1614	down
Ylangene	Terpenoids	1.2708	0.4471	−1.1614	down
Di-epi-.alpha.-cedrene-(I)	Terpenoids	1.2708	0.4471	−1.1614	down
2-Epi-.alpha.-funebrene	Terpenoids	1.2708	0.4471	−1.1614	down
3,4-Dimethylcyclohexanol	Alcohol	1.4363	0.4457	−1.1658	down
7-Oxabicyclo[4.1.0]heptane, 1-methyl-4-(1-methylethenyl)-	Terpenoids	1.2685	0.4433	−1.1736	down
Limonene oxide, cis-	Terpenoids	1.2685	0.4433	−1.1736	down
Limonene oxide, trans-	Terpenoids	1.2685	0.4433	−1.1736	down
(+)-(*E*)-Limonene oxide	Terpenoids	1.2685	0.4433	−1.1736	down

As shown in Table 4, a comparison between the JD and JD-L groups revealed 40 compounds with significant differences. These comprised five alcohols, one aromatic compound, one phenolic compound, one nitrogen-containing compound, one halogenated hydrocarbon, two ethers, five acids, ten terpenes, one ketone, six heterocyclic compounds, and seven esters. Among these, the most pronounced increase was observed for the terpene (1*S*)-(+)-3-carene, which was 37.34 times more abundant in the JD-L group than in the JD group. The most notable reduction occurred in hydantoin, a nitrogen-containing compound, with its level in the JD-L group measuring 0.4973 times that in the JD group. (1*S*)-(+)-3-Carenene has demonstrated efficacy in inhibiting specific bacterial strains (Shu et al., 2020; Tang et al., 2021). It may also influence the microbial community balance during fermentation and the stability of wine. Furthermore, it contributes floral, fruity, and herbaceous notes to the wine (Marques et al., 2023). As a nitrogenous compound, hydantoin generally enhances flavors in baked goods, nuts, and caramel (Le Menn et al., 2017; Yan et al., 2021). It can serve as a precursor in reactions such as the Maillard reaction and Strecker degradation, thereby generating other flavor-active compounds (Shibamoto, 2019). Thus, hydantoin may directly or indirectly impact the functional properties of food.

**Table 4:** Top 40 compounds showing differences in volatile flavor compounds between the JD and JD-L groups.

Compounds	Class I	VIP	Fold_Change	Log2FC	Type
(+)-3-Carene	Terpenoids	1.5230	37.3434	5.2228	up
Bicyclo[4.1.0]hept-2-ene, 3,7,7-trimethyl-, (1S-cis)-	Terpenoids	1.5230	37.3434	5.2228	up
(+)-4-Carene	Terpenoids	1.5230	37.3434	5.2228	up
3-Carene	Terpenoids	1.5230	37.3434	5.2228	up
Butanoic acid, 3-methyl-, butyl ester	Ester	1.0239	35.0930	5.1331	up
Octadecane, 1-chloro-	Halogenated hydrocarbons	1.7677	26.3805	4.7214	up
Indole, 3-methyl-	Heterocyclic compound	1.0613	24.6165	4.6216	up
1H-Indole, 2-methyl-	Heterocyclic compound	1.0613	24.6165	4.6216	up
Butanenitrile, 4-(methylthio)-	Ether	1.6502	24.4955	4.6144	up
Butanoic acid, decyl ester	Ester	1.1017	24.3818	4.6077	up
2-Oxooctanoic acid	Acid	2.0108	23.2677	4.5403	up
2,7-Octadiene-1,6-diol, 2,6-dimethyl-	Terpenoids	1.0613	22.7610	4.5085	up
2,7-Octadiene-1,6-diol, 2,6-dimethyl-, (*Z*)-	Terpenoids	1.0613	22.7610	4.5085	up
Hexadecanoic acid, 3-hydroxy-, methyl ester	Ester	1.2649	20.5842	4.3635	up
1H-Indene, 2,3-dihydro-4-propyl-	Aromatics	1.0818	16.4465	4.0397	up
Butanoic acid, octyl ester	Ester	1.3254	15.4753	3.9519	up
Diethyl Phthalate	Ester	1.3733	12.9231	3.6919	up
Furan, tetrahydro-2-(3-phenylpropyl)-	Heterocyclic compound	1.0686	11.4301	3.5148	up
Benzoic acid, 4-methoxy-, methyl ester	Ester	1.1657	10.6194	3.4086	up
Butane, 1,1-diethoxy-3-methyl-	Ether	1.0495	9.6869	3.2760	up
Hydantoin	Nitrogen compounds	1.3537	0.4973	−1.0078	down
2,5-Dimethylfuran-3-thiol	Heterocyclic compound	1.8884	0.4927	−1.0211	down
3-Thiophenethiol	Alcohol	1.6268	0.4818	−1.0536	down
2-Thiophenethiol	Alcohol	1.6268	0.4818	−1.0536	down
Octanoic acid, 2-hexenyl ester, (*E*)-	Ester	1.4632	0.4808	−1.0564	down
Octanedioic acid	Acid	1.4147	0.4798	−1.0594	down
Bergamotol, *Z*-.alpha.-trans-	Terpenoids	1.5788	0.4786	−1.0631	down
*p*-Hydroxyphenyl methyl carbinol	Alcohol	1.4129	0.4701	−1.0888	down
2-Buten-1-ol, 2-ethyl-4-(2,2,3-trimethyl-3-cyclopenten-1-yl)-	Alcohol	1.4774	0.4696	−1.0904	down
Levomenol	Terpenoids	1.4007	0.4688	−1.0928	down
2-Hydroxymethylbenzimidazole	Heterocyclic compound	1.4348	0.4687	−1.0932	down
3-Buten-2-one, 4-phenyl-	Ketone	1.5906	0.4682	−1.0948	down
.beta.-Pinene	Terpenoids	1.6985	0.4670	−1.0986	down
Bicyclo[3.1.1]heptane, 6,6-dimethyl-2-methylene-, (1*S*)-	Terpenoids	1.6985	0.4670	−1.0986	down
1,4-Methano-1H-Cyclopropa[*d*]pyridazine, 4,4a,5,5a-tetrahydro-6,6-dimethyl-, (1.alpha.,4.alpha.,4a.alpha.,5a.alpha.)-	Heterocyclic compound	1.4898	0.4645	−1.1061	down
4-Ethyloctanoic acid	Acid	2.0447	0.4645	−1.1063	down
4-Octanol	Alcohol	1.6145	0.4635	−1.1094	down
Hexanoic acid	Acid	1.8582	0.4624	−1.1127	down
Butanoic acid, 3-hydroxy-	Acid	1.3342	0.4570	−1.1296	down
4,6-Dichloro-guaiacol	Phenol	1.4727	0.4516	−1.1470	down

Based on the differential volatile compounds identified, KEGG pathway enrichment analysis was conducted, as presented in Fig. 7, which displays the top 20 pathways ranked by *P*-value. The horizontal axis indicates the Rich Factor for each pathway, and the vertical axis lists the pathway names in ascending order of *P*-value. The color of each dot corresponds to the *P*-value, with redder colors indicating greater enrichment significance, while the dot size represents the number of differential volatile compounds associated with each pathway. As illustrated in Fig. 7, phenylpropanoid biosynthesis and secondary metabolite biosynthesis were among the pathways associated with differential volatile compounds in all three comparisons, namely JD-H vs. JD, JD-D vs. JD, and JD-L vs. JD. Phenylpropanoid-related metabolism is an important biochemical process in grapes and wine and is associated with the formation of phenolic compounds, including flavonoids and anthocyanins (Eremia et al., 2023; Qin et al., 2023). These compounds contribute to wine color, astringency, antioxidant properties, and storage stability (Sánchez-Ortiz et al., 2024). Volatile phenols and other aroma-related compounds may also be associated with phenylpropanoid-related metabolism and can contribute to the aromatic characteristics of wine (Šikuten et al., 2025; Yang et al., 2024). Secondary metabolite-related pathways are likewise associated with a wide range of aroma compounds, including higher alcohols, esters, terpenes, ketones, and aldehydes (Romano et al., 2022; Sieiro-Sampedro et al., 2019). However, many volatile aroma compounds represent downstream or end products and may originate from multiple biochemical or non-biological processes. Therefore, the KEGG enrichment results should be interpreted as indicating potential pathway associations rather than direct evidence of pathway activation or unique microbial origin. Overall, these results suggest that co-fermentation with different non-Saccharomyces yeasts was associated with distinct patterns of volatile compounds related to several metabolic pathways.

**Fig. 7:**
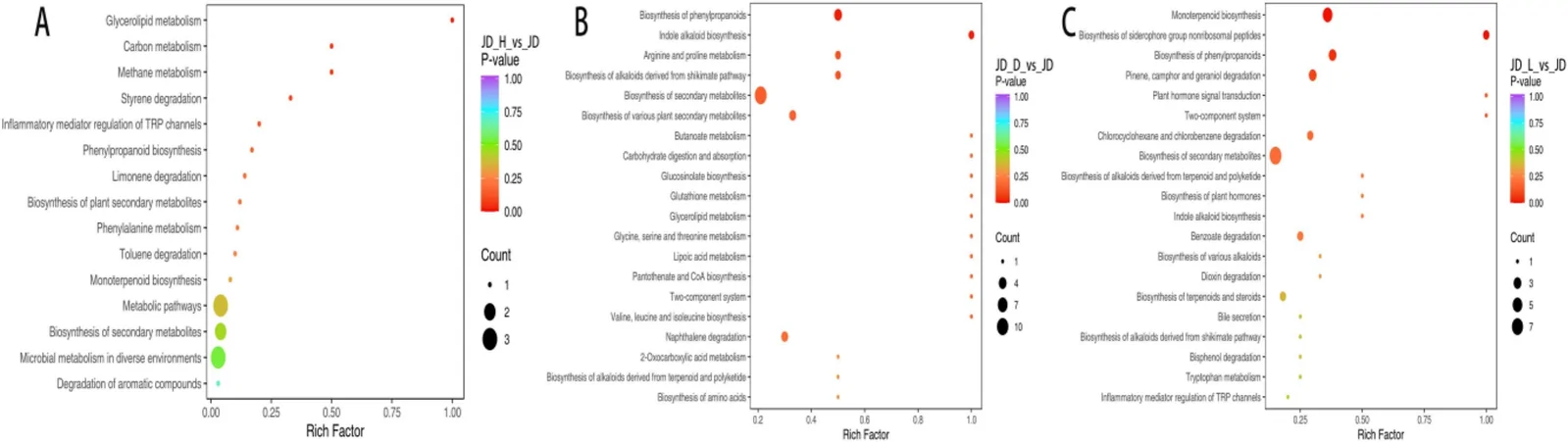
Musalais Difference Abundance Score Plot.

The metabolic pathways compared between the JD-H and JD groups are presented in Fig. 7-A. Pathways associated with glycerol metabolism, carbon metabolism, methane metabolism, styrene degradation, and the regulation of inflammatory mediators by TRP channels were enriched in the JD-H group. Among the three groups, the microbial metabolic pathways in diverse environments exhibited the greatest number of differentially enriched metabolites. These findings suggest that the JD-H treatment was associated with changes in pathways related to carbohydrate utilization and glycolysis.The enrichment of pathways associated with styrene degradation indicates that the fermentation environment may host microorganisms able to degrade or transform these undesirable flavor precursors or by-products, thus contributing to improved wine flavor (dos Santos Júnior et al., 2023).

The metabolic pathways compared between the JD-D and JD groups are presented in Fig. 7-B.Pathways associated with butyrate metabolism, carbohydrate digestion and absorption, glucosinolate biosynthesis, glutathione metabolism, and glycine, serine, and threonine metabolism showed relatively stronger enrichment in the JD-D group.Likewise, pathways involved in thioctic acid metabolism, pantothenic acid and coenzyme A biosynthesis, and the biosynthesis of valine, leucine, and isoleucine also showed greater enrichment in this group. The two groups exhibited numerous differences in the number of metabolites associated with phenylpropanoid biosynthesis and secondary metabolite production. These pathways are involved in metabolizing various sulfur-containing compounds, amino acids, and B vitamins. Notably, glutathione serves as a key antioxidant, which enhances the alcohol's antioxidant capacity and helps prevent oxidative browning. It also safeguards cellular proteins and DNA against oxidative damage, thereby promoting the survival and function of microorganisms under adverse conditions (Kou et al., 2022).The metabolism of glycine, serine, threonine, and branched-chain amino acids, including valine, leucine, and isoleucine, provides precursors for key flavor compounds such as higher alcohols and esters (Lezaeta et al., 2018). These pathway associations suggest possible changes in carbon utilization and the formation of intermediates related to subsequent metabolic pathways and flavor formation(Chen et al., 2023). These compounds significantly enhance the fruity and floral notes in wine. Overall, these processes reveal that microorganisms not only survive during fermentation but also dynamically modulate their metabolic activities in response to the fermentation environment, ultimately shaping the distinctive sensory profile of the wine (Liang et al., 2023; Liang et al., 2023).

The metabolic pathways identified in the comparison between the JD-L and JD groups are presented in Fig. 7-C. The most significantly enriched pathways included those associated with iron carrier-mediated non-ribosomal peptide biosynthesis, plant hormone signal transduction, two-component systems, biosynthesis of terpenes and polyketide alkaloids, plant hormone biosynthesis, and indole alkaloid biosynthesis. Additionally, multiple differentially enriched metabolites were linked to pathways involving monoterpenoid biosynthesis, phenylpropanoid biosynthesis, degradation of pinene, camphor, and geraniol, benzoic acid degradation, biosynthesis of various alkaloids, and dioxin degradation. These pathway associations may reflect microbial responses to nutrient availability and environmental conditions during fermentation.The biosynthesis and degradation of branched-chain amino acids, terpenes, phenylpropanoids, and diverse alkaloids directly or indirectly contribute to the formation of complex flavor compounds in fermented beverages, substantially influencing the sensory quality of these products (Fariña et al., 2012; Yu et al., 2024).

### 3.6 Sensory evaluation

The compounds with the top 50 rOAV values among those analyzed in Musalais rOAV studies are listed in Table 5. In all four fermentation groups, the three compounds with the highest rOAV values (rOAV >2000) were 5-ethyl-3-hydroxy-4-methyl-2(5H)-furanone (a ketone), α,α,4-trimethyl-3-cyclohexen-1-methanethiol (an alcohol), and β-vanillin (a terpene). 5-ethyl-3-hydroxy-4-methyl-2(5H)-furanone has aroma characteristics reminiscent of candies, sweetness, strawberries, and pineapples (Api et al., 2024). In the brewing of Musalais wine, the Maillard reaction between sugars and amino acids, especially during grape juice concentration and natural fermentation, serves as a key mechanism for the formation of 5-ethyl-3-hydroxy-4-methyl-2(5H)-furanone (Zhu et al., 2019). Therefore, this compound may contribute substantially to the intense caramel-like aroma of Musalais. In addition, α,α,4-trimethyl-3-cyclohexen-1-methanethiol acts as a key free thiol, contributing rich aromatic notes of blackcurrant, passion fruit, and citrus fruits characteristic of Muscat varieties (Carlin et al., 2022).β-Ionone is primarily derived from the enzymatic or acidic degradation of carotenoids in grape ingredients (Liang et al., 2021; Wang et al., 2022). This compound contributes to Muscat's characteristic woody and floral aromas (Xie et al., 2023). The synergistic interaction of these three compounds is key to the distinctive flavor and superior quality of Muscat. Furthermore, aldehydes such as (E)-4-nonenal and (E)-2-hexenal, together with esters like 2-phenylethyl 3-methylbutyrate and ethyl tert-butylacetate, impart fresh floral and fruity nuances to the wine. These elements serve to balance the deeper caramel and savory notes, ensuring the wine remains vibrant and avoids becoming overly heavy or flat.

**Table 5:** Top 50 compounds with the highest odor activity values according to Musalais's rOAV method.

Compounds	Class I	JD	JD-H	JD-D	JD-L	Contribution to the flavor profile
2(5H)-Furanone, 5-ethyl-3-hydroxy-4-methyl-	Ketone	26,271.60	24,596.67	27,052.03	25,950.96	Caramel aroma, jam-like sweetness, and nutty flavors
3-Cyclohexene-1-methanethiol, .alpha.,.alpha.,4-trimethyl-	Alcohol	20,650.57	17,071.60	24,439.21	22,996.73	Aroma of baking, with a slight fruity scent
3-Buten-2-one, 4-(2,6,6-trimethyl-1-cyclohexen-1-yl)-	Terpenoids	2278.85	2363.46	2823.39	2431.61	The fragrance of wood and flowers
Benzenemethanethiol	Alcohol	352.61	317.19	481.81	441.05	Smoky, charred aroma
Butanoic acid, 3-methyl-, 2-phenylethyl ester	Ester	282.60	266.95	328.17	275.55	Chrysanthemums, roses, the scent of apples, and the fragrance of honey
4-Nonenal, (*E*)-	Aldehyde	278.16	182.70	710.17	308.18	The scent of grass and oils
1-Nonen-3-one	Ketone	196.35	225.90	181.37	223.51	The smell of mushrooms and soil
1-Octen-3-one	Ketone	114.77	79.88	53.53	74.14	The smell of mushrooms and soil
t-C4H9CH2C(*O*)OCH2CH3	Ester	111.08	178.78	1048.27	456.05	Fresh fruit flavors, a hint of alcohol, and ester notes
Pentanal, 3-methyl-	Aldehyde	93.58	109.98	448.86	165.02	Almond, nuts, and the aroma of oils
trans-3,5-Diethyl-1,2,4-trithiolane	Heterocyclic compound	90.27	92.79	113.65	104.09	Sauce-flavored and pickled flavors
trans-.beta.-Ionone	Terpenoids	79.76	82.72	98.82	85.11	Floral fragrance, woody fragrance, sweet fragrance
Pyrazine, 2-methoxy-3-(1-methylpropyl)-	Heterocyclic compound	79.05	56.90	74.84	26.20	Nuts, soil, and the aroma of coffee
(Z,Z)-3,6-Nonadienal	Aldehyde	75.68	72.61	74.51	73.07	Cucumbers, watermelons, and aquatic plants with a delicate fragrance
2-Hexenal, (E)-	Aldehyde	64.66	64.67	64.56	64.68	The scent of green grass, leaves, and green apples
Non-8-enal	Aldehyde	60.02	57.42	58.99	57.62	The scent of grass and oils
o-Chloroaniline	Amine	52.52	36.07	128.77	55.20	The scent of medicine and the aroma of smoke
trans,cis-2,6-Nonadien-1-ol	Alcohol	43.80	42.06	50.71	44.61	Cucumbers, watermelons, with a delicate and refreshing flavor
Dodecanenitrile	Nitrogen compounds	41.77	39.01	47.32	40.54	Fat, waxiness, and the scent of citrus peels
2-Methylisoborneol	Terpenoids	37.70	35.07	39.19	37.20	Camphor, pine wood, mint scent, and the aroma of wood
2-Nonenal, (*E*)-	Aldehyde	25.48	23.41	23.93	24.81	The scent of grass and oils
3-Mercapto-3-methylbutyl formate (ester)	Ester	21.76	24.78	1.89	77.41	Grilled meat, scallion aroma, sauce flavor
2-Nonenal	Aldehyde	20.39	18.73	19.14	19.85	The scent of grass and oils
3-Mercaptohexyl acetate	Ester	16.24	15.25	16.54	15.91	Grilled meat, scallion aroma, sauce flavor
Thiazole, 2,5-diethyl-	Heterocyclic compound	16.20	16.36	15.50	16.52	Nuts, meaty flavors, and the aroma of baking
Benzaldehyde, 4-methoxy-	Aldehyde	15.23	13.93	14.41	14.59	Vanilla, almonds, and sweet spices
2-Hexenal	Aldehyde	11.72	11.73	11.71	11.73	The fragrance of green grass
1,3,5-Trithiane	Heterocyclic compound	10.99	5.75	6.26	6.53	Roasted nuts and meaty flavors
Phenylethyl Alcohol	Alcohol	10.48	7.16	25.27	11.65	Roses, honey, and sweet floral scents
Phenol, 2,4-dibromo-	Phenol	10.28	10.77	12.44	8.67	The scent of medicine and the aroma of smoke
Germacrene D	Terpenoids	7.91	7.58	9.29	7.83	Wooden, herbaceous, with the scent of turpentine
1-Butanol, 3-methyl-, acetate	Ester	5.83	7.97	41.27	19.37	Bananas, pears, and a refreshing fruity aroma
2-Buten-1-one, 1-(2,6,6-trimethyl-1,3-cyclohexadien-1-yl)-, (*E*)-	Terpenoids	5.82	4.87	6.44	5.48	Rose, honey fragrance, and tropical fruit scents
Phenol, 4-chloro-	Phenol	5.82	4.49	3.60	4.70	The scent of medicine and the aroma of smoke
2,4-Undecadienal	Aldehyde	5.31	4.64	4.37	4.34	The scent of grass and oils
Phenol, 2-methoxy-	Phenol	5.29	5.07	5.19	5.09	Smoked flavors, vanilla, herbal scents, cloves
4-Heptanone	Ketone	4.62	4.79	4.75	4.75	Oils, cheeses, and subtle fruity flavors
Pyrazine, 2-ethyl-3,5-dimethyl-	Heterocyclic compound	4.61	11.62	9.25	7.57	The aroma of roasted nuts, cocoa, and coffee
6-Nonenal, (E)-	Aldehyde	4.45	4.28	6.55	4.83	The scent of grass and oils
Benzeneacetaldehyde	Aldehyde	4.45	1.86	1.60	2.67	Rose fragrance, floral scent, sweet fruit aroma, honey scent
Benzene, (2-isothiocyanatoethyl)-	Ester	4.29	4.28	6.18	4.51	Mustard, spicy flavor, and the aroma of vegetables
Butanoic acid, 3-methyl-, ethyl ester	Ester	4.05	4.06	3.91	3.90	The scent of apples and pineapples
2-octenal	Aldehyde	4.05	3.55	4.89	4.06	Oils and grassy aromas
Pentanoic acid, 3-methyl-, ethyl ester	Ester	3.24	2.66	11.64	11.57	Sweet fruit aroma, with a hint of alcohol
Hexanoic acid, ethyl ester	Ester	3.00	1.68	2.88	2.82	Pineapple, bananas, and the aroma of sweet wine
Nonanoic acid	Acid	2.86	1.64	2.11	1.70	Fat, waxy texture, with a slightly acidic aroma
Cyclohexanone, 2,2,6-trimethyl-	Ketone	2.39	3.05	2.78	2.89	Wood fragrance, camphor, and a slight sweet scent
2-Undecenal, *E*-	Aldehyde	2.38	1.78	8.76	1.47	The scent of grass and oils
1-Butanol, 2-methyl-, acetate	Ester	2.33	3.19	16.51	7.75	Fresh fruit flavors, a hint of alcohol, and ester notes
2-Methoxy-4-vinylphenol	Phenol	2.28	1.14	4.04	4.52	Scent of cloves, vanilla, and caramel sweetness

Fig. 8 illustrates the differences in flavor compounds between Musalais samples and the control group, presenting the ten most dominant sensory flavors for each comparison. In the case of JD-H versus JD (Figs. 8-A and 8-D), four principal sensory flavors were identified, comprising 15 sweet, 13 fruity, 11 floral, and 10 nutty metabolites. Terpene derivatives, such as (E,E)-2,6-dimethyl-2,4,6-octatriene, contribute fruity, floral, and subtle citrus notes to Musalais. Fruit-related metabolites, including ethyl benzoate and 1-(2,6,6-trimethyl-1,3-cyclohexadienyl)-2-butenone, contribute cooked apple, rose, honey, dried fruit, tea, and berry-spice aromas, thereby enhancing the aromatic complexity and sensory pleasantness of Musalais. In the comparison between JD-L and JD (Figs. 8-B, 8-E), 19 sweet, 18 herbaceous, 16 nutty, and 16 woody metabolites were identified. Propylcyclohexane imparts a subtle hydrocarbon-like aroma, while microbial-derived esters such as butyric acid 2-methylpropyl ester and hexyl acetate contribute pleasant fruity and floral notes. Additionally, 2-methoxy-3-(1-methylpropyl)pyrazine and other pyrazine compounds provide distinctive plant-associated aromas. In the comparison of JD-D versus JD (Figs. 8-C, 8-F), 50 sweet, 48 fruity, 31 herbaceous, and 26 floral metabolites were identified. Notably, butyl 3-methylbutyrate imparts tropical fruit and sweet notes, whereas 1-phenylethyl butyrate offers elegant rose and ripe fruit aromas. Nevertheless, this group exhibited a high abundance of herbaceous metabolites. An overly pronounced grassy odor could disrupt the overall flavor balance, resulting in a monotonous and less desirable sensory profile for Musalais (Zhu, Zhang, et al., 2021; Zhu et al., 2023).

**Fig. 8:**
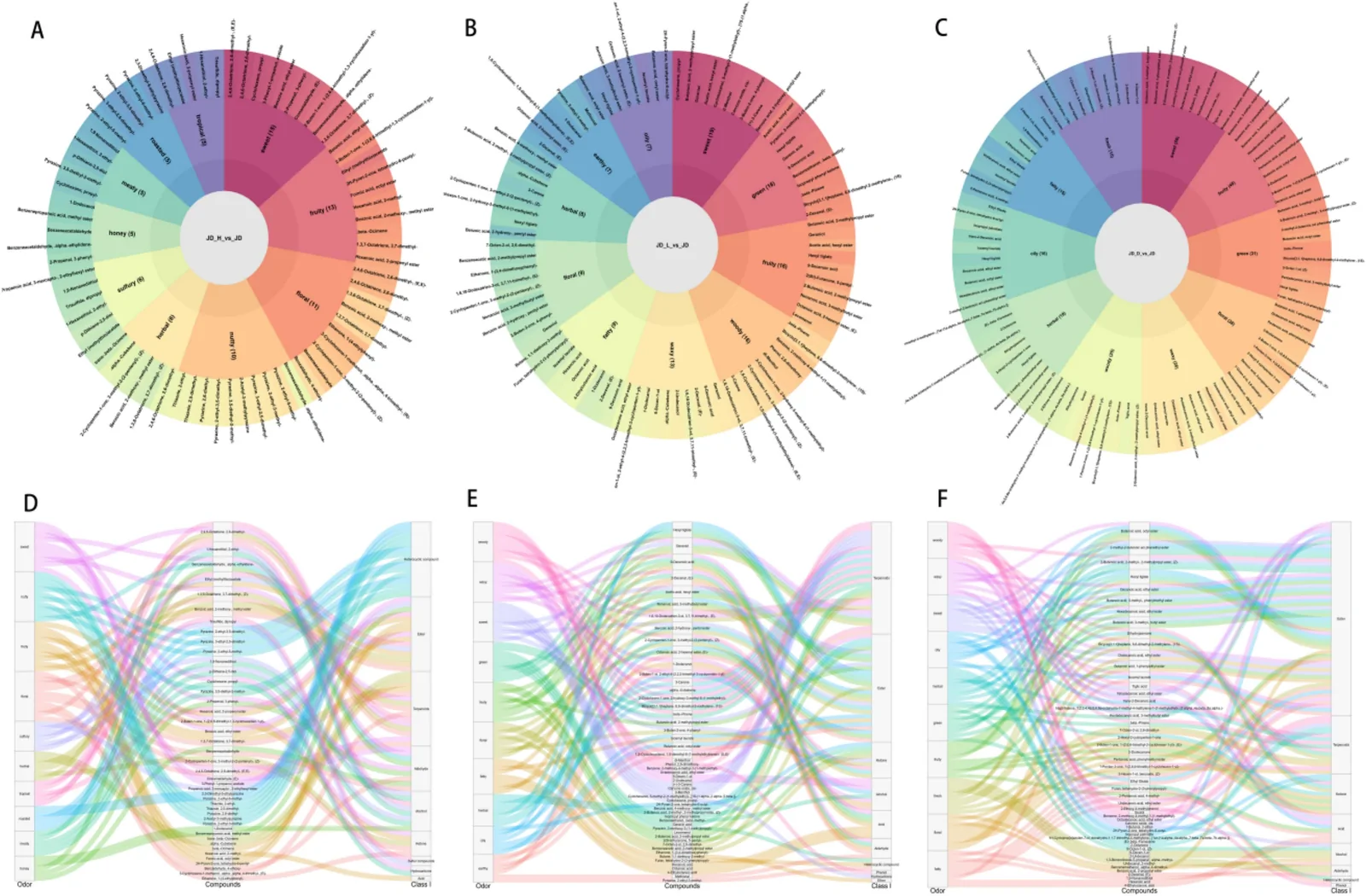
Musalais Comparative Difference Flavor Compound Flavor Wheel and Sangi Diagram.

Sensory evaluation was conducted across four fermentation groups, with the following sensory scores: JD (86.10 ± 0.03), JD-H (88.10 ± 0.84), JD-D (86.40 ± 0.80), and JD-L (91.20 ± 0.61). The overall quality ranking was JD-L > JD-H > JD-D > JD. JD-L exhibited substance concentrations similar to those in the JD group, without excessive accumulation, thereby preserving the traditional sensory characteristics of Musalais wine and achieving excellent flavor balance. KEGG pathway analysis further showed enrichment of pathways associated with monoterpenoid biosynthesis and phenylpropanoid metabolism in JD-L. The observed enrichment pattern may be associated with the release and transformation of terpene-related aroma compounds; however, the underlying enzymatic mechanisms were not directly examined in the present study.This group was rich in terpenoid derivatives; esters imparted the wine with characteristic fruity and floral notes, along with subtle nutty and woody nuances. Its aroma profile was complex yet harmonious, devoid of grassy or off-odors, aligning with its superior sensory score. JD-H received a slightly lower score despite containing comparable compound levels to JD. Its metabolic pathways were primarily enriched in phenylpropanoid biosynthesis and carbon metabolism. The JD-H group showed enrichment of pathways associated with carbon metabolism together with changes in acetate-related volatile compounds, which may have contributed to its fruity and floral aroma characteristics. However, less active terpene metabolism limited terpene release, leading to lower intensity and persistence of floral notes compared with JD-L. A slightly higher proportion of grassy compounds also reduced aroma softness and harmony, resulting in a moderate sensory score. JD-D ranked third, exhibiting the most active metabolism and significantly higher concentrations of flavor substances than JD, including a 1.4-fold increase in aldehyde content, along with vigorous metabolism of higher alcohols, esters, and branched-chain amino acids. Its total sweet and fruity metabolites were the highest of all groups. Typically, aldehydes are reduced to alcohols during late fermentation; however, insufficient alcohol dehydrogenase activity or premature termination of fermentation can lead to aldehyde accumulation, resulting in a harsh and pungent flavor. Excessive aldehydes, combined with elevated levels of higher alcohols and amines, masked the natural fruity and floral aromas, creating a disordered and inharmonious fragrance profile. Although JD-D exhibited the highest abundance of volatile compounds, it failed to achieve a favorable flavor balance, which led to a lower sensory score compared with JD-L and JD-H. Similarly, Arslan et al. (2018), Luo et al. (2023), and Li, Huang, et al. (2024) reported improved aroma or sensory quality following non-Saccharomyces co-fermentation. Consistent with these studies, JD-L achieved the highest sensory score in the present study. Notably, JD-D showed the highest overall volatile abundance but a lower sensory score than JD-L, suggesting that sensory quality depended more on the balance and composition of volatile compounds than on their total abundance.

Fermented exclusively with Saccharomyces, the JD group received the lowest sensory evaluation score. While it completed standard alcoholic fermentation and generated fundamental flavor compounds, it was unable to synthesize terpenes and other complex aromatic substances. KEGG pathway analysis indicated underdevelopment in key biosynthetic pathways, such as those for phenylpropanoids and monoterpenoids. Consequently, the JD sample exhibited only a simple alcoholic taste and basic ester aroma, lacking the distinctive floral, fruity, and nutty notes characteristic of Musalais wines, and was rated lowest in overall sensory quality.

## 4 Conclusions

This study compared pure Saccharomyces JD fermentation with co-fermentation using three non-Saccharomyces yeasts (HX, DE, LZ) in Muscat grapes.The three non-Saccharomyces yeasts exerted distinct strain-dependent effects on Musalais fermentation, volatile metabolite profiles, and sensory characteristics.By 96 h, the alcohol contents of all fermentation groups had reached the typical range for Musalais, suggesting that 96 h may represent a more appropriate practical fermentation endpoint for controlling alcohol accumulation, although fermentation was still ongoing at this stage.Among the 1866 volatile compounds detected, 1826 were shared across all fermentation groups, indicating that co-fermentation primarily altered the relative abundance of the common volatile metabolites rather than fundamentally changing their overall occurrence.Consistent with previous studies, non-Saccharomyces co-fermentation modified alcohol, acid, and volatile compound profiles, but the magnitude and direction of these effects were strain-dependent. JD-D showed the greatest flavor divergence from pure JD. KEGG enrichment analysis suggested that differential volatile compounds were associated with pathways including phenylpropanoid biosynthesis and secondary metabolite biosynthesis. Sensory preference ranked JD-L > JD-H > JD-D > JD. The highest sensory score of JD-L, despite JD-D showing the greatest overall volatile abundance, further indicates that sensory quality was related to the composition and balance of volatile compounds rather than their total abundance alone. *Non-Saccharomyces* yeasts enhanced aroma complexity, offering a strategy to improve Musalais quality.Overall, this study reveals that the influence of non-Saccharomyces yeasts on Musalais is highly strain-dependent and demonstrates associations among mixed-culture fermentation, volatile metabolite remodeling, aroma-active compounds, and sensory quality.These findings broaden the understanding of mixed-culture fermentation in Musalais and provide a theoretical basis for the targeted selection of non-Saccharomyces strains to modulate its flavor characteristics.Overall, the findings provide a basis for future scale-up evaluation and the potential application of selected non-Saccharomyces yeasts in Musalais production.

The following are the supplementary data related to this article.

**Supplementary Fig. S1:**
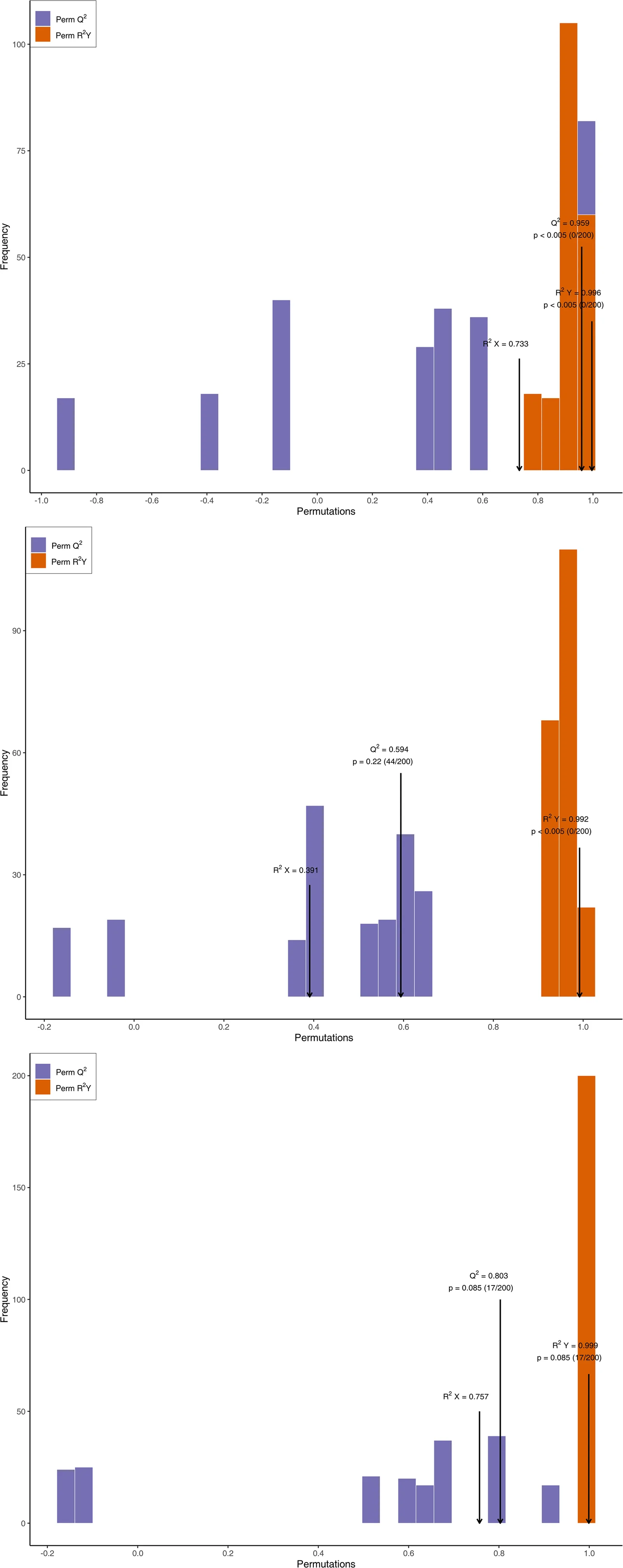
Permutation test results for the multivariate discriminant analysis models. The purple and orange bars represent the distributions of permuted Q² and R²Y values, respectively. Arrows indicate the corresponding statistics of the original models. The permutation test was performed with 200 permutations to evaluate model robustness and potential overfitting.

Supplementary material 1 Detailed identification information for volatile compounds detected in Musalais samples, including compound identifiers, chemical names, molecular formulas, and associated analytical parameters.

Supplementary material 2 Odor thresholds and relative odor activity values (rOAVs) of volatile compounds detected in Musalais samples under different fermentation treatments.

Supplementary material 3 Volatile compounds exhibiting non-overlapping occurrence patterns among the four fermentation treatments (JD, JD-H, JD-D, and JD-L).

## CRediT authorship contribution statement

**Rui Tang:** Writing – review & editing, Writing – original draft, Data curation, Conceptualization. **Tengteng Ge:** Writing – review & editing, Writing – original draft, Data curation, Conceptualization. **Buhailiqiemu Abudureheman:** Supervision, Funding acquisition. **Yu Zhang:** Writing – original draft, Formal analysis. **Jianlin Zhang:** Supervision, Project administration. **Lin Chen:** Writing – review & editing, Formal analysis. **Liang Wang:** Writing – review & editing, Supervision. **Jingyang Hong:** Writing – review & editing, Project administration, Funding acquisition.

## Declaration of competing interest

The authors declare that they have no known competing financial interests or personal relationships that could have appeared to influence the work reported in this paper.

## Data Availability

Data will be made available on request.
